# SVM-Based Classification of sEMG Signals for Upper-Limb Self-Rehabilitation Training

**DOI:** 10.3389/fnbot.2019.00031

**Published:** 2019-06-04

**Authors:** Siqi Cai, Yan Chen, Shuangyuan Huang, Yan Wu, Haiqing Zheng, Xin Li, Longhan Xie

**Affiliations:** ^1^Shien-Ming Wu School of Intelligent Engineering, South China University of Technology, Guangzhou, China; ^2^A^*^STAR Institute for Infocomm Research, Singapore, Singapore; ^3^The Third Affiliated Hospital, Sun Yat-sen University, Guangzhou, China

**Keywords:** surface electromyography, support vector machine, rehabilitation robot, upper limb, motion pattern recognition

## Abstract

Robot-assisted rehabilitation is a growing field that can provide an intensity, quality, and quantity of treatment that exceed therapist-mediated rehabilitation. Several control algorithms have been implemented in rehabilitation robots to develop a patient-cooperative strategy with the capacity to understand the intention of the user and provide suitable rehabilitation training. In this paper, we present an upper-limb motion pattern recognition method using surface electromyography (sEMG) signals with a support vector machine (SVM) to control a rehabilitation robot, ReRobot, which was built to conduct upper-limb rehabilitation training for post-stroke patients. For poststroke rehabilitation training using the ReRobot, the upper-limb motion of the patient's healthy side is first recognized by detecting and processing the sEMG signals; then, the ReRobot assists the impaired arm in conducting mirror rehabilitation therapy. To train and test the SVM model, five healthy subjects participated in the experiments and performed five standard upper-limb motions, including shoulder flexion, abduction, internal rotation, external rotation, and elbow joint flexion. Good accuracy was demonstrated in experimental results from the five healthy subjects. By recognizing the model motion of the healthy side, the rehabilitation robot can provide mirror therapy to the affected side. This method can be used as a control strategy of upper-limb rehabilitation robots for self-rehabilitation training with stroke patients.

## Introduction

Stroke is the leading cause of adult disability around the world (Burton et al., 2017), with upper-limb motor impairments being the main factor influencing the quality of life in stroke survivors (Stinear et al., 2017). Repetitive motor training on movement has a notable curative effect on the restoration of arm function in stroke patients, and the patients' degree of recovery is positively influenced by treatment intensity (Steven et al., 2006; Gittler and Davis, 2018). Conventionally, stroke patients usually rehabilitate with the assistance of therapists. However, the involvement of therapists is challenging because rehabilitation training is a time-consuming and labor-intensive process. Many stroke survivors experience upper-limb impairment with few rehabilitation opportunities due to a lack of rehabilitation therapists. Robot-assisted therapy devices, which can provide the affected arm with high intensity and repetitive treatment, have been increasingly used in rehabilitation training and can potentially enhance upper-limb functional recovery in stroke survivors (Yoo and Kim, 2015; Veerbeek et al., 2017).

Various rehabilitation robotic devices have been developed for upper-limb training in stroke patients. Among them, MIT-Manus (Krebs et al., 1999) was one of the first systems to be developed and can provide stroke survivors with plane movements. Furthermore, MIME (Lum et al., 2006), GENTLE/s (Coote et al., 2008), T-WREX (Domien et al., 2011), and NEREBOT (Stefano et al., 2014) were proposed to permit three-dimensional exercise training for patients with impaired arms.

Different control strategies have been developed and applied to rehabilitation robots for the recovery of the affected arm. Motion parameters of the patient's arm are one of the major inputs in the rehabilitation robot's control system. Many types of mechanical inputs, such as switches (e.g., Aubin et al., 2013; Artz et al., 2015), force sensors (e.g., Diftler et al., 2014), and computer vision (e.g., Taati et al., 2012), have been used as feedback in the controllers of rehabilitation robots. The surface electromyography (sEMG) signal, which is composed of the action potentials from groups of muscle fibers, is one of the major sources of information about neural control and can reflect the degree of activity of the muscles (Yang et al., 2016). During the rehabilitation training of the upper limb, sEMG signals can be captured, interpreted, and used as input for the control algorithms of rehabilitation robots (Rosen et al., 2001; Kiguchi and Hayashi, 2012; Peternel et al., 2016). Considering that rich motor control information and the user's intention can be detected from sEMG signals, the sEMG-based control scheme is one of the most appropriately suited approaches for upper-limb rehabilitation robots (Singh et al., 2014).

However, the sEMG signal is affected by many factors and is not stable, which can lead to low accuracy in recognizing patient motion intentions. Some studies have shown that machine learning techniques can be employed for classifying different tasks and improving the robustness and accuracy of the identification and classification of arm movements through the exploitation of sEMG signals (Lucas et al., 2008; Young et al., 2013; Suberbiola et al., 2015). The SVM algorithm is a well-established technique to learn how to classify new data starting from a collection of classified events and has been widely applied in machine learning problems (Vapnik, 1995; Suykens et al., 2015) and sEMG processing (Song et al., 2007) because of its simplicity and robustness. With the determination of a few additional tuning parameters, SVM solutions are characterized by a convex quadratic optimization problem (Platt, 1999). Considering that the availability and quality of sEMG signals can vary from patient to patient, it is difficult to obtain a large number of training samples. SVM is suitable for solving learning tasks where the number of attributes is large relative to the number of training examples (Suykens et al., 2002).

Aimed at developing a control strategy for upper-limb rehabilitation robots with the capacity to understand the intention of the patients and provide the corresponding rehabilitation training, this paper proposed an sEMG-based control framework based on SVM classifiers for intention identification of the upper limb. The control strategy was applied to the upper-limb rehabilitation robot, ReRobot, to perform the rehabilitative exercise training. The motion of the patient's healthy side is first recognized from the measured and processed sEMG signals, and then the ReRobot assists the affected side in conducting the corresponding rehabilitation therapy. Based on the developed sEMG-based control strategy, self-rehabilitation training in stroke patients can be conducted.

## Methods

### Data Collection

Many stroke patients have trouble moving their upper limbs on the affected side, and they must receive much rehabilitation training to recover motion ability (Merletti et al., 1999). Stroke patients often show abnormal shoulder motor ability, so shoulder rehabilitation actions, including shoulder forward flexion, shoulder level adduction, and shoulder level abduction (Chen and Zhou, 2015), should be carried out.

**Figure 1:**
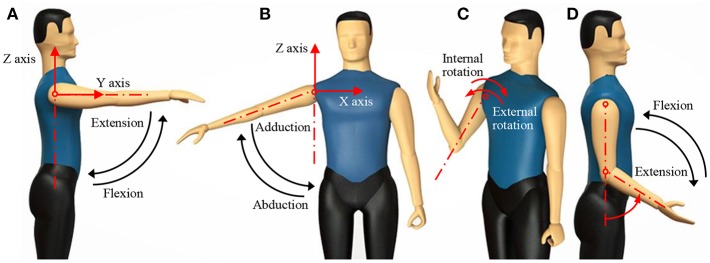
Human upper-limb motions. **(A)** Shoulder flexion; **(B)** shoulder abduction; **(C)** shoulder internal rotation and external rotation; **(D)** elbow flexion.

The coordinates were defined where the coronal axis of the patient is the *X*-axis, the sagittal axis is the *Y*-axis, the vertical axis is the *Z*-axis, and the acromioclavicular joint is the origin of the coordinates. Five motions were used to test the performance of this model, including shoulder flexion, abduction, internal rotation, external rotation, and elbow joint flexion, as shown in Figure 1.

Five healthy subjects (*N* = 5, age 25 ± 4 years, body mass 70 ± 5 kg, height 174 ± 6 cm, all male and all right-handed) participated in the experiments. All subjects gave their informed consent before participation. The experimental procedures were conducted in accordance with the Declaration of Helsinki and approved by the Ethic Board of Medical School, South China University of Technology. Each subject performed five repetitions in accordance with five standard motions. Since stroke patients are mostly elderly people with lower motion abilities on their healthy side compared to young adults, the designed motions were imitated as movements of elderly stroke patients on their healthy side.

For each test, the test subject did not carry weight, and the movement lasted for 1–2 s. After the end of each movement, the subject took at least 1 min to rest to prevent muscle fatigue. There are many muscles involved in the movement of the shoulder and elbow joints. Muscles play two roles in the movement process: proximal stability and distal activity. In this paper, eight superficial muscles involved in the distal shoulder and elbow movements were selected as monitoring objects. To ensure safety, patients controlled the emergency stop of the equipment according to their own comfort level. In this paper, the patient's clenched fist is used as the emergency stop action, and the flexor radialis is used as the detection channel for the emergency stop action. Therefore, a total of nine muscles were selected as test objects. The sEMG signals of nine muscles in the upper limb were acquired, including the middle deltoid, anterior deltoid, pectoralis major, biceps brachii, brachioradial muscle, ulnar flexor carpal, trapezius, posterior deltoid, and triceps brachii. The first eight muscles were used to evaluate how the muscle works while the signal changes. The ulnar flexor carpal played a role in the subject's self-initiation of the safety protection mechanism. When the subject felt uncomfortable during the test, he or she could stop the robot by clenching his or her fist and activating the ulnar flexor.

**Figure 2:**
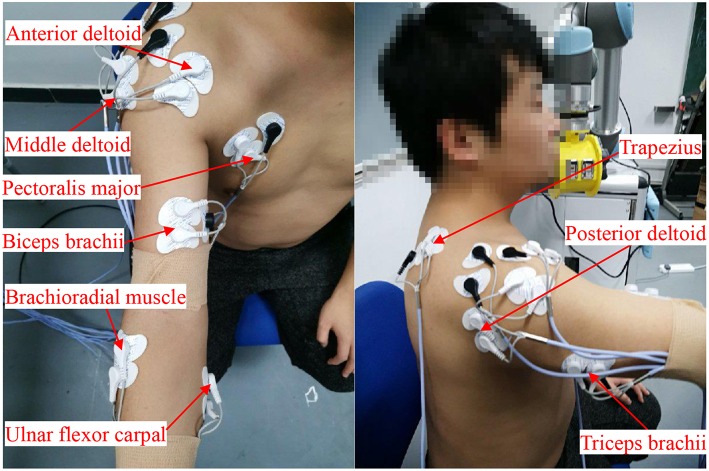
Locations of the sEMG electrodes: middle deltoid, anterior deltoid, pectoralis major, biceps brachii, brachioradial muscle, ulnar flexor carpal, trapezius, posterior deltoid, and triceps brachii. Written informed consent was obtained from the individual in this image for the publication.

**Figure 3:**
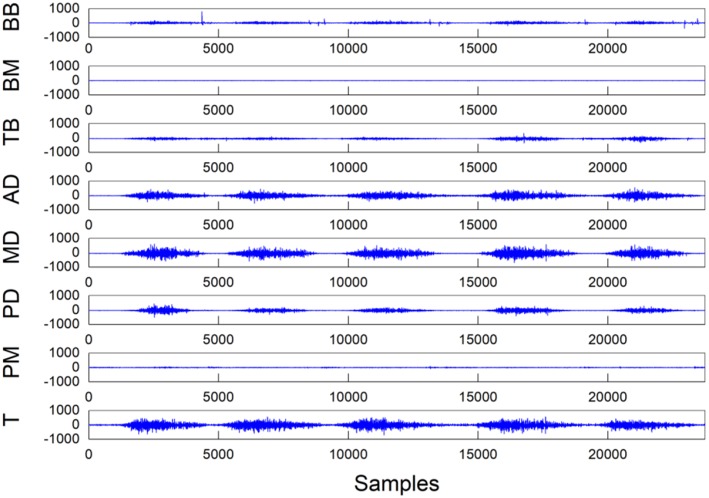
Raw experimental data recorded during a single trial of shoulder abduction from one of the five individuals. The unit of the *Y* coordinate is μV. (T, trapezius; PM, pectoralis major; PD, posterior deltoid; MD, middle deltoid; AD, anterior deltoid; TB, triceps brachii; BM, brachioradial muscle; BB, biceps brachii).

Figure 2 shows the experimental setup. A 16-channel sEMG acquisition instrument with 1-kHz sampling frequency in each channel was used. Each channel was related to a three-channel differential electrode. After the muscle was disinfected by alcohol, the electrodes were placed along the direction of the muscle abdomen with an interval of 2 cm. Figure 3 shows the raw sEMG signals of the eight muscles without preprocessing from one of the five healthy individuals.

### Data Processing

#### Preprocessing

sEMG signals are easily disturbed by the external environment in the acquisition process. Motion artifacts, baseline offset, and power frequency interference may all lead to distortion of the sEMG signals, which leads to poor classification accuracy (Chen et al., 2015). Data preprocessing methods of baseline correction, 20–500 Hz bandpass filter, power frequency filter (50 Hz notch), full-wave rectification, and amplitude normalization were carried out to improve the signal-to-noise ratio (SNR) of the sEMG signal. All filters used in this paper are fourth-order Butterworth filters.

#### Signal Segmentation

Although the intensity of the sEMG signal detected in each channel is different in the different movements, the signals show good synchronization (Zhang and Zhou, 2012): if the related muscles did not contract, then the sEMG signal showed a stable low-amplitude signal before the test; in contrast, the signal changed dramatically in the course of executing the action. The characteristic of this type of signal was that the signal could be segmented by a sample entropy algorithm (Liu and Zhou, 2013). The sample entropy algorithm is an efficient and time-consuming algorithm that can avoid the signal deviation caused by self-matching. Therefore, we adopted the sample entropy algorithm for data segmentation.

In the experiments, the action signals of eight channels were collected, and the muscle signal for the sample entropy analysis was from the sum of the eight-channel signals, which is:

(1) $$\begin{array}{r} \text{sEMG}(\text{t})\;=\;\sum_{i=1}^{M}\;sEMG_{i}\left(t\right) \end{array}$$

where *M* is the total number of channels, sEMG_*i*_(*t*) is the *tth* value of channel *i*, and *sEMG* is the sum of all channel signals.

**Figure 4:**
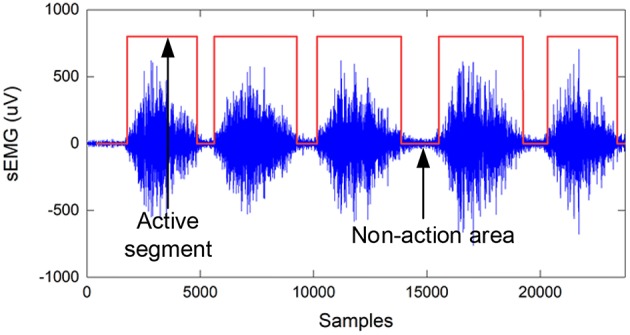
Teacher sample labels based on the sample entropy algorithm, where the label value of the active segment is 1, and the non-action area is 0.

The sample entropy can be calculated as follows:

(2) $$\begin{array}{r} \text{SampEn }\left(m,r,L\right)=-\ln\left[\frac{B^{m+1}\left(r\right)}{B^{m}\left(r\right)}\right] \end{array}$$

where *m* is the dimension of the sEMG signal, *r* is the similar tolerance, *L* is the length of the muscle signal, and *B*^*m*^ (*r*) is the probability of the two signal sequences matching *m* points. In this study, we set *m* = 2 and *r* = 0.25^*^σ, where σ is the standard deviation of the sEMG signal.

(3) $$s\left(n\right)\;=\;\{\begin{array}{cc} 0,\; & |SampEn\;<\;d\\ 1,\; & |SampEn\;\geq \;d \end{array}$$

where *d* (*d* = 0.6) is the threshold and *s*(*n*) is the judgment function of the EMG signal. When *s*(*n*) = 1, it is the effective part of the action; when *s*(*n*) = 0, it is the invalid part of the action, as shown in Figure 4.

#### Feature Extraction and Classification

Because of the short-term stationarity of sEMG signals, the signals need to be divided into frames. To prevent spectrum leakage, window functions should be used for interception. Compared with window functions such as the rectangular window and triangular window, the Hanning window has the characteristics of fast side lobe attenuation and is suitable for non-stationary signals. Therefore, this paper adopts a Hanning window for framing. Hanning windows with window lengths ranging from 30 to 300 ms (Chowdhury et al., 2013) were used to extract the characteristics of the sEMG signals. To ensure the implementation of the system and the stability of the classification, 128 ms was selected as the window length, and the sliding step size was 64 ms. The root mean square (RMS), fourth-order autocorrelation factor, wavelength, variance, absolute mean, and short-term energy of each window in each channel are calculated as follows:

(4) $$\begin{array}{rcl} {\text{RMS}}_{kj} & = & \sqrt{\frac{1}{N}\sum_{i=1}^{N}{\left(sEMG_{ij}\right)}^{2}} \end{array}$$

(5) $$\begin{array}{rcl} {\text{VAR}}_{kj} & = & \frac{1}{N}\sum_{i=1}^{N}\left(sEMG_{ij}-\bar{sEMG_{j}}\right) \end{array}$$

(6) $$\begin{array}{rcl} {\text{MAV}}_{kj} & = & \frac{1}{N}\sum_{i=1}^{N}|sEMG_{ij}| \end{array}$$

(7) $$\begin{array}{rcl} {\text{SSI}}_{kj} & = & \sum_{i=1}^{N}{\left(sEMG_{ij}\right)}^{2} \end{array}$$

where *k* represents the *kth* window and *j* is the *j*th channel.

#### Support Vector Machine

Support vector machine (SVM) is a machine learning method based on statistical learning theory. The characteristic behavior of SVM is to construct a high-dimensional hyperplane for small samples and non-linear models and to classify samples by calculating the maximum distance of training data points on the hyperplane (Ma et al., 2004). Due to the physical limitations of stroke patients, the sample size of the data that can be collected is small. In small-sample model training, SVM has advantages of higher stability and fewer training parameters (Raczko and Zagajewski, 2017). Therefore, SVM is a better choice than a neural network. The equation solved by the SVM algorithm after the Lagrange operator can be expressed as:

(8) $$\{\begin{array}{c} \min\;\frac{1}{2}\;{\left\|w\right\|}^{2}\;+\;C\sum_{i=1}^{N}\;\xi i\\ s.t.y_{k}(w•x_{k}\;+\;b)\;\geq \;1\;-\;\xi_{i},\;k\;=\;1...N \end{array}$$

where (**x**_**k**_, *y*_*k*_) represents the training data of the *kth* window. ξ_*i*_ is a slack variable, which represents the magnitude of the classification error.

The radial basis kernel function can be expressed as (Chung et al., 2003):

(9) $$\begin{array}{r} \kappa \left({\text{x}}_{\text{i}},{\text{x}}_{\text{j}}\right)=\exp\left\{\;\frac{||{\text{x}}_{\text{i}}-{\text{x}}_{\text{j}}|{|}^{2}}{2\delta^{2}}\right\} \end{array}$$

**Figure 5:**
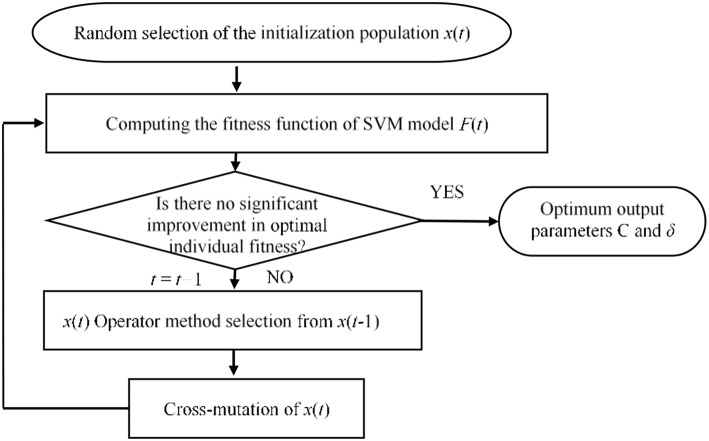
Parameter optimization process based on a genetic algorithm.

The penalty factor *C* and kernel function parameter δ are the main parameters that affect the performance of the model. Therefore, while training the model, the penalty factor *C* and kernel function parameter δ should be optimized. To optimize parameters *C* and δ of the model, the genetic optimization algorithm is used. The accuracy of the time series prediction is selected as the fitness function. The optimization steps of SVM parameters based on the genetic algorithm are shown in Figure 5.

To test the feasibility of the genetic optimization algorithm, simulations were carried out using MATLAB. Five healthy subjects (*N* = 5, age 25 ± 4 years, body mass 70 ± 5 kg, height 174 ± 6 cm, all male and all right-handed) were selected for shoulder flexion, abduction, pronation, and elbow flexion. Each action was performed five times for data classification and recognition.

The default value of MATLAB is *C* = 1; σ = 1/num_features_, where num_features_ is the number of features. In this paper, num_features_ = 40. The value after optimization is *C* = 0.4579 and σ = 371.6339. The influence of the parameter optimization is significant. Compared with the default value, the optimized value has higher classification accuracy. When the default parameters are used, the classification accuracy is 78.53%. After parameter optimization, the classification accuracy reached 94.18%.

## Upper-Limb Rehabilitation Robot Platform

### Robot System

**Figure 6:**
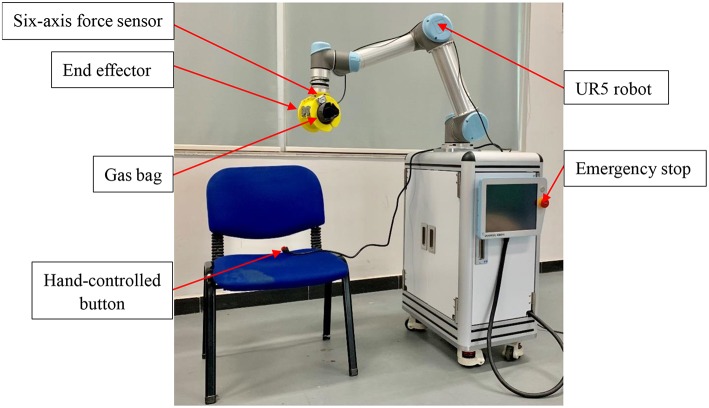
The ReRobot system. The system is set up to support seated rehabilitation training in 3D space, and the UR5 robot is the main system used.

The ReRobot is a rehabilitation robot platform developed to permit training of the upper limb in three-dimensional space, as shown in Figure 6. The platform is set up to support and guide the movement of the affected arm using a UR5 robot arm. UR robotic arms are lightweight, fast, easy to program, flexible, and safe robotic arms with 6 DOF (Kebria et al., 2017). The configuration can provide positioning and orientation to a patient's upper limb in the training tasks. The transmission control protocol/internet protocol (TCP/IP) was used to communicate with the robot and the MATLAB user interface. After attaching to the forearm of a stroke patient, ReRobot focuses on the rehabilitation exercise of the shoulder and elbow joints in accordance with the range of movement of the human arm, including shoulder flexion/extension, shoulder abduction/adduction, shoulder internal/external rotation, elbow joint flexion/extension, and forearm supination/pronation. The rehabilitation data, e.g., data that include the sEMG, forces, velocities, and positions, are used for analysis and ensure the safety of subject is collected in real time during the exercises. To ensure a comfortable fixation to the patient's arm, a gas bag is used as the buffer device at the attachment point with the human arm.

Safety is an important issue in the ReRobot therapy system. The safety system consists of several components, including an emergency stop switch, force sensor stops, a hand-controlled switch, and an sEMG signal stop. The emergency stop button, which is held by the experimenter, cuts power to the robot and shuts down all systems. The six-axis force sensor located at the robot arm measures interaction forces generated during the tasks. If the interaction force is abnormal, the power of the system would be automatically cut off. The hand-controlled switch, which is held by the subject, stops the movement of the robot. In the event of any abnormality, the robot stops and the patient's arm can be easily removed from the end of the ReRobot by a freely actuating mechanism and removable ends.

### Control Scheme Based on the SVM Classification of sEMG Signals

**Figure 7:**
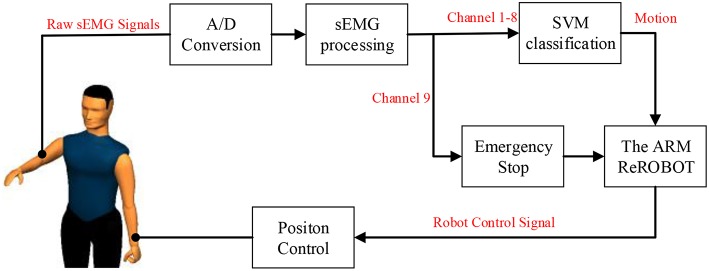
Control structure for robot-assisted exercise training based on the SVM classification of sEMG signals.

The control system of the ReRobot system is intended to develop a human–machine interface (HMI) that is able to activate the device as soon as the patient's motion intention is detected. By using the SVM system, the upper-limb movements of the healthy side can be detected and classified automatically through sEMG signals, and the ReRobot will then assist the impaired arm with that movement. The control scheme based on the SVM classification of sEMG signals was thus established, as shown in Figure 7.

This platform acquires the test subject's sEMG signals of the upper limb on the healthy side, and the preprocessed EMG signals from one to eight channels are used for upper limb motion recognition using the SVM classification method. The recognized action label signals are sent to the robot for motion calculation, and then the robot actuates the subject's affected arm with the position control method to perform the corresponding actions based on a presupposed trajectory.

When the patient feels discomfort in his or her arm or muscle abnormalities during the test, the whole system can be safely stopped by patient through the first clenching motion, and the sEMG signal from channel 9 is sent to the manipulator. At the same time, there is an emergency stop button in the test subject's hand throughout the test process to ensure safety.

### Experiment and Results

**Figure 8:**
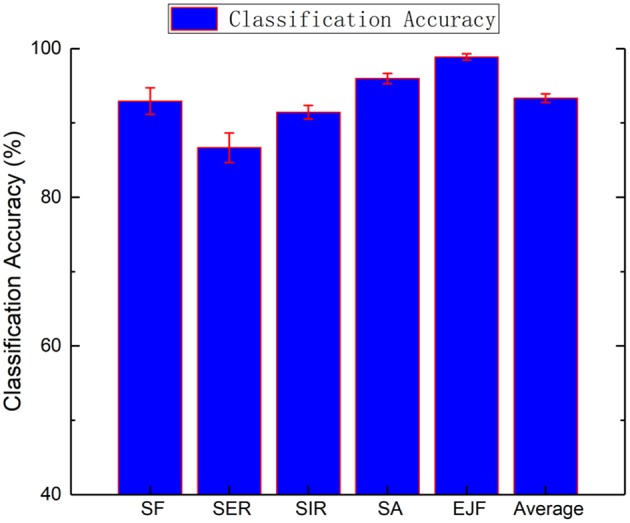
Mean recognition accuracies for the five healthy subjects (SF, shoulder flexion; SER, shoulder external rotation; SIR, shoulder internal rotation; SA, shoulder abduction; and EJF, elbow joint flexion).

**Figure 9:**
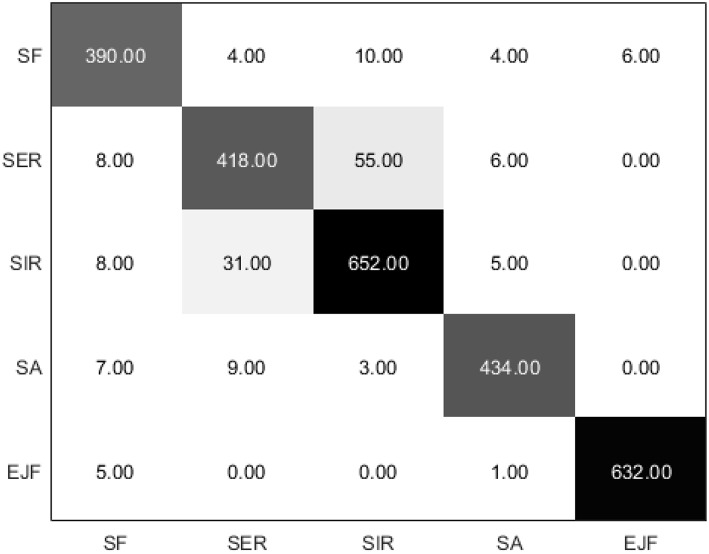
Chaotic matrix of the five healthy subjects (the diagonal value of the matrix is the correct number of classifications, while the non-diagonal value is the wrong number of classifications).

To test the feasibility of the upper-limb motion recognition method based on the SVM classification, simulations were carried out using MATLAB. Five movements of five normal people were collected 20 times. After processing feature extractions and label recognition of the collected data, 10 five-fold cross-validations were performed. The results are shown in Figure 8. The classification accuracy of each action is as follows: average recognition rate, 93.34 ± 0.59%; shoulder flexion, 92.95 ± 1.78%; shoulder external rotation, 91.44 ± 0.91%; shoulder internal rotation, 86.67 ± 1.98%; shoulder abduction, 95.98 ± 0.70%; and elbow flexion, 98.89 ± 0.42%. The chaotic matrix of one of the classifications is shown in Figure 9. The misclassification rate of shoulder internal rotation and shoulder external rotation is high. The main reason is that the muscles involved in the two movements have a high coincidence, so they are easily confused. However, the overall recognition rate is high, so the system can be used in the actual operation of the experimental platform.

Based on the chaotic matrix, three accuracy metrics (precision, recall, and F1-score) can be obtained (Sokolova and Lapalme, 2009). Precision describes the accuracy of the detection. Recall is the detection rate, which refers to how well the target objects are detected without being missed. The F1-score combines the precision and recall and provides a single measure of quality that is easy for end-users to understand. The precision, recall, and F1-score of SVM algorithm in classifying five different motions, including shoulder flexion, shoulder external rotation, shoulder internal rotation, shoulder abduction, and elbow joint flexion, were calculated to evaluate the performance, as shown in Table 1. The elbow joint flexion was detected with excellent performance (F1-score = 0.991), followed by shoulder abduction (F1-score = 0.961), shoulder flexion (F1-score = 0.921), and shoulder external rotation (F1-score = 0.881). The SVM-based classifier generally classified well and the average F1-score of five types of motions was 0.9368.

**Table 1:** Classification performance—five types of motions.

**Motion**	**Precision**	**Recall**	**F1-score**
Shoulder flexion	0.933	0.942	0.938
Shoulder external rotation	0.905	0.858	0.881
Shoulder internal rotation	0.906	0.937	0.921
Shoulder abduction	0.964	0.958	0.961
Elbow joint flexion	0.991	0.991	0.991

**Figure 10:**
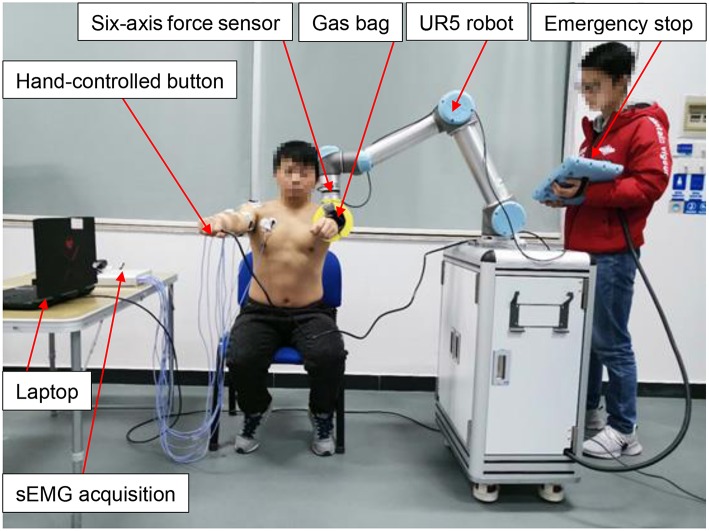
Experiments for sEMG signal acquisition and SVM-based classification during rehabilitation exercises from the healthy side of the subject. Written informed consents were obtained from the individuals in this image for the publication.

Five subjects (*N* = 5, age 25 ± 4 years, body mass 70 ± 5 kg, height 174 ± 6 cm, all male and all right-handed) participated in the rehabilitation training experiments, and all five subjects were able to complete robot-assisted voluntary exercises, as shown in Figure 10. Since these five subjects are all right-handed people, the sEMG signals of their right arms were acquired and processed. Based on the SVM classification method, their motion intentions were analyzed and used as input into the control schema of ReRobot. Thus, ReRobot can understand the desired movement of the subjects and facilitate its execution, thus providing active support to their left arms. A total of 100 actions were performed in the actual test, and 92 actions were correctly identified using the SVM-based method. The robot arm successfully assisted the subject's arm with recognized movements. Multiple security guarantees ensure the safety of the subjects in this process. The experimental results showed that SVM-based classification achieved good accuracy in rehabilitation training.

To verify the effectiveness of the safety switch based on the sEMG signal, a total of 25 tests of safety stops based on sEMG signals were performed. ReRobot had its cut power during each test, which proved the feasibility of using sEMG signals as an emergency stop in the rehabilitation system.

The experimental results showed that SVM-based classification can provide good accuracy in upper-limb motion pattern recognition and enabled patients to choose actions actively for rehabilitation. It is possible to use sEMG signals as an emergency stop button in the upper-limb rehabilitation system to ensure safety.

## Conclusions

In this paper, we investigated the feasibility of SVM classifiers for intention identification of the upper limb from sEMG signals. A new human–machine interface for self-rehabilitation training with stroke patients was developed. The upper-limb rehabilitation robot, ReRobot, could adequately understand the desired upper-limb movement and facilitate its execution, thus providing active support to the impaired arm. Experiments with the ReRobot showed that the SVM classification based on sEMG signals can provide good accuracy in upper-limb motion pattern recognition when a time-dependent multifeature set was used.

In future research, this method to extract upper-limb intention from sEMG signals will be tested by experiments with stroke patients. The application of this classifier to upper-limb rehabilitation robots will be implemented to achieve successful clinical verification.

## Ethics Statement

This study was carried out in accordance with the recommendations of SCUT Research Ethics Guidelines and Researcher's Handbook, Ethic Board of Medical school, South China University of Technology with written informed consent from all subjects. All subjects gave written informed consent in accordance with the Declaration of Helsinki. The protocol was approved by the Ethic Board of Medical school, South China University of Technology.

## Author Contributions

SC, YC, and LX contributed conception and design of the study. SH carried out the experiments. SC and YC performed the formal analysis and methodology part. SC wrote the first draft of the manuscript. YC, SH, YW, HZ, XL, and LX wrote sections of the manuscript. All authors contributed to manuscript revision, read and approved the submitted version.

### Conflict of Interest Statement

The authors declare that the research was conducted in the absence of any commercial or financial relationships that could be construed as a potential conflict of interest.
